# Combined Amylin/GLP-1 pharmacotherapy to promote and sustain long-lasting weight loss

**DOI:** 10.1038/s41598-019-44591-8

**Published:** 2019-06-11

**Authors:** Claudia G. Liberini, Kieran Koch-Laskowski, Evan Shaulson, Lauren E. McGrath, Rachele K. Lipsky, Rinzin Lhamo, Misgana Ghidewon, Tyler Ling, Lauren M. Stein, Matthew R. Hayes

**Affiliations:** Department of Psychiatry, Perelman School of Medicine, University of Pennsylvania, 19104 Philadelphia, PA USA

**Keywords:** Obesity, Obesity

## Abstract

A growing appreciation of the overlapping neuroendocrine mechanisms controlling energy balance has highlighted combination therapies as a promising strategy to enhance sustained weight loss. Here, we investigated whether amylin- and glucagon-like-peptide-1 (GLP-1)-based combination therapies produce greater food intake- and body weight-suppressive effects compared to monotherapies in both lean and diet-induced obese (DIO) rats. In chow-maintained rats, systemic amylin and GLP-1 combine to reduce meal size. Furthermore, the amylin and GLP-1 analogs salmon calcitonin (sCT) and liraglutide produce synergistic-like reductions in 24 hours energy intake and body weight. The administration of sCT with liraglutide also led to a significant enhancement in cFos-activation in the dorsal-vagal-complex (DVC) compared to mono-therapy, suggesting an activation of distinct, yet overlapping neural substrates in this critical energy balance hub. In DIO animals, long-term daily administration of this combination therapy, specifically in a stepwise manner, results in reduced energy intake and greater body weight loss over time when compared to chronic mono- and combined-treated groups, without affecting GLP-1 receptor, preproglucagon or amylin-receptor gene expression in the DVC.

## Introduction

Obesity affects more than one-third of individuals in the world, creating an enormous health and economic burden, yet effective non-invasive treatments are limited^1,2^. The failure of behavioral interventions to produce sustained weight loss in an overwhelming majority of cases underscores the importance of identifying novel therapeutic strategies to promote and sustain reductions in food intake and body weight. As energy homeostasis involves innumerous interactions among redundant neural mechanisms^3–5^, it is reasonable to consider that treating a complex disease such as obesity may require combinatorial therapies that target multiple energy balance-regulating pathways. However, identifying the right combinatorial therapeutic strategies has proven challenging. Ideally, an effective combination drug therapy for treating obesity would enhance the magnitude and temporal duration of weight loss but would also engage a set of partially-overlapping, complementary neural circuits to mechanistically suppress feeding. To this end, the amylin and glucagon-like peptide-1 (GLP-1) hormonal systems have been independently identified as promising targets for pharmacotherapy intervention^6–11^.

While the appetite suppressive effects of amylin and GLP-1 receptor agonists are known to involve direct actions in the brain^6,7,9,10,12–14^, uncovering the neuroanatomical site(s) of action and the physiological mechanisms that mediate the anorectic response remains an area of intense research focus. In the hindbrain, the dorsal-vagal-complex (DVC), collectively comprised of the nucleus tractus solitarius (NTS), area postrema (AP) and dorsal motor nucleus of the vagus (DMV), mediates the anorectic effects of both amylin and GLP-1 receptor agonists^15–19^. As amylin and GLP-1 target different populations of neurons in the DVC^20^, this suggests that combination drug therapies affecting both systems may provide a greater magnitude and sustainable degree of weight loss compared to monotherapies due to a reduced likelihood of tachyphylaxis^21–23^. Indeed, a critical barrier that the obesity research field must overcome is to find pharmacotherapies that can break-through the plateau of weight loss that occurs with every existing FDA-approved treatment for obesity^1,24–28^.

Due in part to the short half-life of endogenous amylin and GLP-1^29,30^, long acting agonists for amylin (*e*.*g*. pramlintide) and GLP-1 receptors (GLP-1R; *e*.*g*. liraglutide) have been developed^21–23,31–35^. Pramlintide and liraglutide are already FDA-approved monotherapies for the treatment of type II diabetes^36^. In addition, peripheral once-daily administration of liraglutide produces a clinically-meaningful degree of body weight loss by reducing energy intake, and as such, liraglutide is also an approved monotherapy for treating obesity^37,38^. Both amylin- and GLP-1-based pharmacotherapies have been separately examined preclinically in the context of combinatorial therapies for obesity treatment with other hormonal systems (*e*.*g;* leptin, glucagon, glucose dependent insulinotropic peptide)^6,39–42^; however, there is a surprising paucity of reports systematically examining the hypothesis that combined amylin and GLP-1 therapies may provide enhanced suppression in food intake and body weight^43,44^. Among the limited work published on GLP-1-amylin combinatorial studies, Bello *et al*.^43^, showed that in non-human primates, injections of the GLP-1R agonist, exendin-4, and the amylin/calcitonin-receptor agonist, salmon calcitonin (sCT), combine to produce a synergistic suppression of food intake. In addition, evidence demonstrate that administration of a chimeric hybrid of the amylin analog davalintide conjugated with the GLP-1 analog exenatide, is beneficial to reduce body weight^44^. However, the exact mechanism of these responses remains unclear. Therefore, here we aim to uncover the behavioral and potential neuroanatomical mechanisms governing the interaction between amylin and GLP-1 signaling in control of energy balance.

## Results

### Combined administration of native amylin and GLP-1 or their receptor analogs enhanced the suppression of food intake and body weight gain compared to individual monotherapies

**Figure 1:**
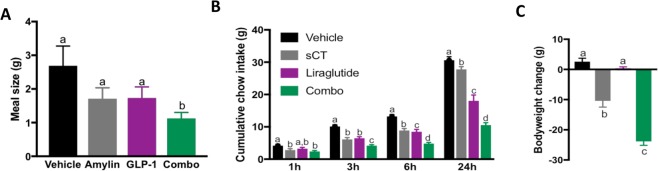
Acute combined treatment of amylin, GLP-1 and their anlogs enhances the suppression of food intake and and body weight gain compared to individual monotherapies. (**A**) Combined administration of native amylin and GLP-1 suppress food intake by decreasing meal size in chow-fed rats (P < 0.05 One-way ANOVA vechile *vs* combo). (**B**) Combined therapy with salmon calcitonin (sCT) and liraglutide sinergistically reduce feeding at 3, 6 and 24 h and (**C**) body weight gain compared to vehicle, sCT and liragliutide alone (P < 0.05 One-way ANOVA). Different letters within each figure significantly differ from each other. Data are expressed as mean ± S.E.M.

To provide a detailed analysis of the behavioral mechanisms mediating the food intake suppressive effects of native amylin and GLP-1 when acutely administered alone or in combination, rats (*n* = 14) were placed in our automated feedometer system with *ad libitum* access to chow and water and meal patterns were objectively analyzed. Animals received a systemic injection of amylin (5 μg/kg), GLP-1 (100 μg/kg), a combination of amylin and GLP-1 or vehicle. Our meal pattern analyses revealed that the combined administration of native amylin and GLP-1 was effective in reducing the size of the first meal to a significantly greater degree than vehicle, amylin alone or GLP-1 alone, suggesting that these hormones act in concert to reduce meal size (P < 0.05, One-way ANOVA; Fig. 1A).

In a separate cohort of chow-maintained rats, the effects of acute administration of an amylin/calcitonin receptor agonist and GLP-1R agonist were examined. Rats (*n* = 14) were acutely injected intraperitoneally (*IP*) with: vehicle, the amylin/calcitonin receptor agonist salmon calcitonin (sCT; 3 μg/kg) alone, the GLP-1R agonist liraglutide (25 μg/kg) alone or a combination of sCT and liraglutide. Cumulative food intake and body weight change were assessed 24 h post-injections. The collective results demonstrate that combined therapy of sCT and liraglutide significantly reduced food intake and body weight compared to vehicle and to a greater degree than the magnitude of food intake and body weight suppression observed after treatment with either mono-therapy alone (Fig. 1B,C). Specifically, individual treatment with the amylin agonist sCT or with the GLP-1R agonist, liraglutide, successfully decreased 24 h body weight change and food intake at 1, 3, 6 and 24 h post-injection (Fig. 1B,C). Acute administration of sCT and liraglutide as a combination therapy significantly suppressed body weight and feeding compared to vehicle and monotherapies at 3, 6 and 24 h post-injection (P < 0.05, One-way ANOVA, Fig. 1B,C). Collectively, our results demonstrate that an acute combinational therapy with amylin and GLP-1R analogs is more effective than individual monotherapies to reduce feeding and body weight gain in lean rats.

### sCT alone and combined with liraglutide treatment increases the number of cFos- activated neurons in the DVC of adult rats

**Figure 2:**
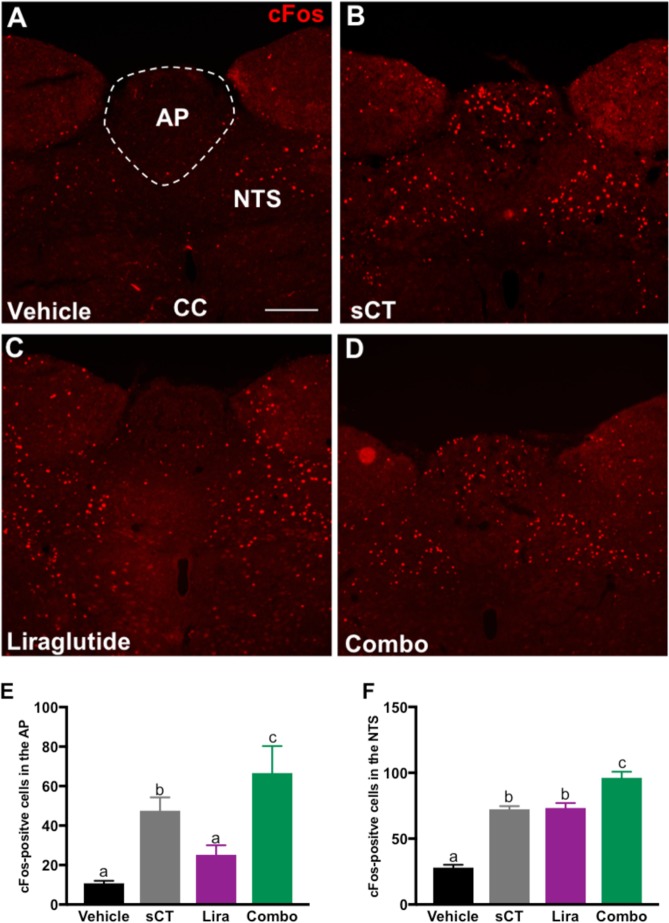
Acute administration of amylin and GLP-1R analogs increases the number of cFos-immuno-positive neurons in the DVC of adult rats. Immunohistochemical staining of cFos-positive cells in DVC sections of (**A**) vehicle, (**B**) sCT (salmon calcitonin), (**C**) liraglutide or (**D**) combo (sCT + liraglutide) treated rats (*n* = 4 per group). (**E**) In the AP, sCT significantly increased the number of cFos-positive cells compared to vehicle, while combo treatment resulted in greater neuronal activation compared to vehicle and individual treatment of sCT and liraglutide (P < 0.01 One-way ANOVA). (**F**) In the NTS of adult rats, acute treatment with sCT and liraglutide alone or in combination, increased the number of cFos-positive cells compared to vehicle (P < 0.01, One-way ANOVA). AP = area postrema, NTS = nucleus tractus solitarious, CC = central canal. Different letters within each figure significantly differ from each other. Data are expressed as mean ± S.E.M. Scale bar 100 um.

In an attempt to identify nuclei within the brain that may offer a complementary set of neural circuits that mediate, at least in part, the combined anorectic effects of amylin and GLP-1 pharmacotherapy, we focused our attention on those nuclei in the DVC given the wealth of literature showing the contribution of this structure to the intake suppressive effects of either amylin- or GLP-1-based monotherapy^6–8,10^. Specifically, we performed c-Fos fluorescent immunohistochemistry on brainstem coronal sections containing the DVC following acute treatment with sCT (3 μg/kg; *IP*), liraglutide (50 μg/kg; *IP*), sCT + liraglutide or vehicle (Fig. 2A–D). Administration of sCT alone or in combination with liraglutide significantly increased the number of c-Fos-labeled neurons in the AP (P < 0.05, One-way ANOVA, Fig. 2E). In the NTS, treatment with sCT or liraglutide alone resulted in an increased number of c-Fos positive cells compared to control, while administration of sCT + liraglutide significantly augmented the number of c-Fos-expressing neurons compared to vehicle or to individual administration of sCT or liraglutide (P < 0.05, One-way ANOVA, Fig. 2F). These results suggest the DVC is an essential hub for mediating the beneficial effect of amylin and GLP-1R analogs combinational therapy.

### A stepwise-regimen of combining amylin pharmacotherapy with liraglutide strongly suppress energy intake and body weight gain in DIO rats

While several studies have described the intake and body weight suppressive effects of liraglutide and sCT administration as monotherapies^21,22,43,45^, a direct comparison of the chronic food intake and body weight suppressive effects of amylin and GLP-1R analogs administered individually or in a combination is still lacking. We therefore aimed to determine whether combined administration of sCT and liraglutide is able to reduce feeding and body weight to a greater extent than either individual monotherapies. As weight loss with any existing pharmaceutical intervention inevitably plateaus due to putative counterregulatory mechanisms, we further hypothesized that the postulated enhanced body weight suppressive effects of combined amylin- and GLP-1R-based pharmacotherapies could be even further enhanced if the drugs were introduced in a stepwise fashion, allowing the physiological systems to adjust to and overcome the tachyphylaxis that occurs with repeated drug treatments^21–23^. Rats were maintained on a free diet-choice of chow and 60% high fat/high sugar diet (HFSD) throughout the experiment. To further exacerbate the onset of obesity (770 g mean body weight at the start of the experiment), rats also received two Nabisco® Golden Oreo cookies per day in addition to *ad libitum* food access. Rats were run in two separate cohorts (*n* = 5–6 per group/wave), where for each experimental test day rats received daily *IP* injections of vehicle, sCT (3 μg/kg), liraglutide (50 μg/kg), a combination of sCT and liraglutide (3 μg/kg + 50 μg/kg) or a stepwise administration of the amylin and GLP-1R analogs (rats received sCT treatment for the first week, liraglutide treatment for the second week, followed by a combination treatment of sCT + liraglutide daily for the remaining two weeks of testing). Cumulative body weight gain and food intake was measured every 24 h for the duration of the experiment.

**Figure 3:**
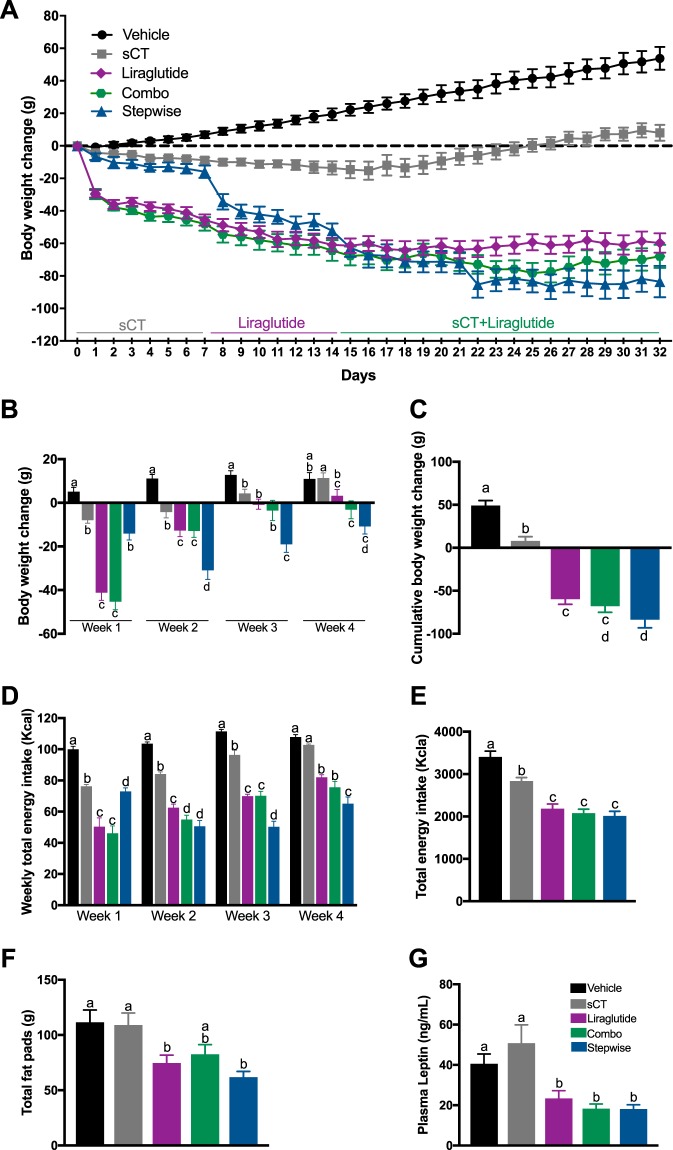
Chronic combination therapy of amylin and GLP-1R analogs results in greater and long-lasting body weight loss and decreased energy intake compared to monotherapies in obese rats. (**A**) Individual and combined treatment with amylin and GLP-1R analogs sCT and liraglutide significantly reduced body weight gain in obese rats (*n* = 10–11 per group; P < 0.05 One-way ANOVA). Stepwise administration (*i*.*e;* first week sCT tretament, second week liraglutide, followed by combo of sCT and liraglutide for the remaining time) of sCT and liraglutide (blue line) resulted in a greater and long-lasing body weight loss compared to vehicle and individual monotherapies. Moreover, amylin and GLP-1R analogs adminstered stewpwise can break the plateau commonly observed with individual monotherapies. (**B**) Weekly body weight loss and (**C**) cumulative body weight change. Combo treatment of sCT and liraglutide, expecially when administered in a stepwise fashion (blue bar) significantly suppressed body weight gain (P < 0.05 One-way ANOVA). Energy intake was recordered daily after *IP* injections of sCT (3 ug/kg), liraglutide (50 ug/kg), combo (3 ug/kg sCT + 50 ug/kg liraglutide), stepwise (week 1 sCT, week 2 liraglutide, week 3 and 4 combo of sCT and liraglutide) or vehicle. Amylin and GLP-1R analogs inejections, individual or combined synergistically decreased (**D**) weekly and (**E**) total cumulative energy intake compared to vehicle (P < 0.05 One-way ANOVA). Stepwise administration of amylin and GLP-1R analogs suppressed weekly energy intake (*e*.*g;* week 2, 3 and 4) compared to vehicle and individual monotherapies (P < 0.05 One-way ANOVA). Liraglutide alone, in combination with sCT or administered stepwise significantly reduced (**F**) adiposity and (**G**) plasma leptin levels compared to vehicle and sCT alone (P < 0.05 One-way ANOVA). Different letters within each figure significantly differ from each other. Data are expressed as mean ± S.E.M.

Chronic treatment with sCT, liraglutide, a combination of both sCT and liraglutide or a stepwise administration of the aforementioned amylin and GLP-1R analogs significantly reduced body weight gain compared to vehicle at weeks 1, 2, 3 and 4 (P < 0.05, One-way ANOVA, Fig. 2B). Starting from week 2, stepwise administration resulted in greater body weight loss compared to vehicle and individual monotherapies. Treatment with sCT, liraglutide, a combination of both sCT and liraglutide or a stepwise administration of sCT and liraglutide, significantly reduced cumulative body weight gain compared to vehicle (P < 0.05, One-way ANOVA, Fig. 3C).

The food intake suppressive effects of chronic amylin and GLP-1R analog treatments were evaluated in rats with *ad libitum* free-choice of chow and HFSD and are expressed in Kcal from food (*n* = 10–11 per diet/drug/group) over the 32 days of treatment following 5 weeks of *ad libitum* diet maintenance. sCT and liraglutide administered either alone, in combination or stepwise significantly suppressed weekly and cumulative energy intake compared to vehicle, while liraglutide alone, combined, or stepwise administered was effective to reduce energy intake compared to sCT and vehicle (P < 0.05, One-way ANOVA; Fig. 3D,E). Stepwise administration of sCT for the first week of treatment, liraglutide for the second week, and a combination of sCT and liraglutide for the remaining treatment period, progressively reduced caloric intake compared to vehicle and monotherapies alone over the entire 32-days treatment period (P < 0.05, One-way ANOVA; Fig. 3D,E). Finally, chronic treatment with liraglutide either alone, in combination with sCT, or administered in a stepwise manner significantly reduced adiposity and plasma leptin levels (P < 0.05, One-way ANOVA; Fig. 2F,G).

Together, our results demonstrate that monotherapies with sCT or liraglutide are effective to reduce body weight gain, with liraglutide treatment resulting in a greater weight loss compared to sCT. The combined treatment of sCT and liraglutide, especially when administered in a stepwise manner, produced greater body weight loss, reduced adiposity and decreased plasma leptin levels compared to individual monotherapies and vehicle in DIO rats. Moreover, the stepwise administration of amylin and GLP-1R analogs was able to break the *plateau* normally observed with individual pharmacotherapies, even when combined from the beginning of treatment, thus supporting the hypothesis that a stepwise-introduction of a combinatorial pharmacological approach at treating obesity is more effective in achieving a sustained and long-lasting degree of weight loss.

### Chronic administration of amylin and GLP-1R analogs does not affect amylin- and GLP-1-related gene expression in the DVC of adult rats

**Figure 4:**
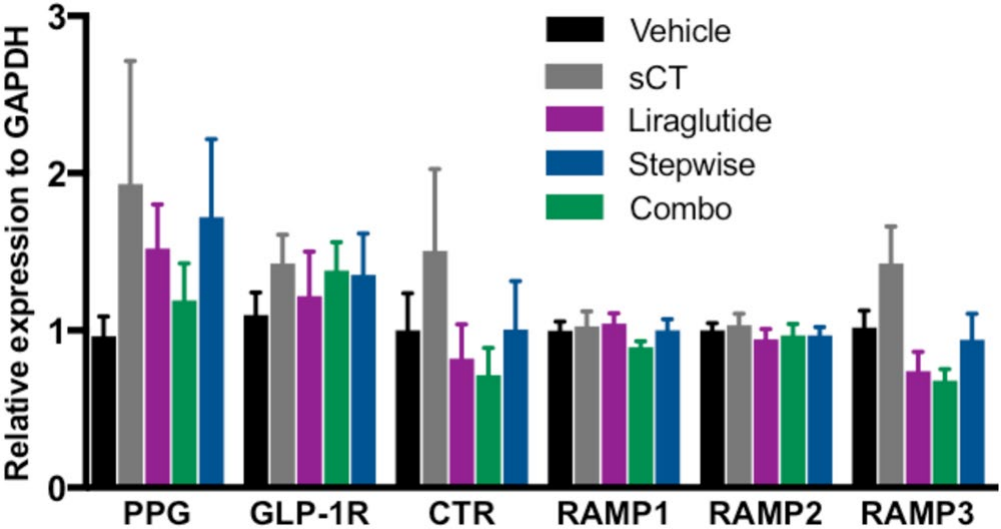
Chronic treatment with amylin and GLP-1R analogs does not affect related gene expression in the DVC of adult rats. Animals (*n* = 10–11 per group) recived daily *IP* injections of sCT (3 ug/kg), liraglutide (50 ug/kg), combo (3 ug/kg sCT+ 50 ug/kg liraglutide), stepwise (week 1 sCT, week 2 liraglutide, week 3 and 4 combo of sCT and liraglutide) or vehicle. Amylin and GLP-1R analogs administration did not affect PPG (pre-proglucagon), GLP-1R, CTR (calcitonin receptor), RAMP (Receptor Activity Modyfing Protein)1, RAMP2 or RAMP3 mRNA expression in the DVC of adult rats. GAPDH was used as endogenous control. Data are expressed as mean ± S.E.M.

To determine whether the profound degree of sustained weight loss observed with chronic administration of amylin and GLP-1R analogs could potentially affect the expression of amylin- and/or GLP-1- ligand/receptor expressing cells in the DVC, we performed qPCR analyses on micro-punched DVC tissue collected from our animals at the termination of the chronic experiment. Our results show that chronic treatment with sCT, liraglutide, a combination of sCT and liraglutide or a stepwise administration of the aforementioned compounds did not affect the regulation of pre-proglucagon (PPG), GLP-1R, calcitonin receptor (CTR), Receptor Activity Modifying Protein (RAMP)1, RAMP2 or RAMP3 mRNA compared to vehicle (Fig. 4). These findings suggest that even in a weight-reduced state from untreated obesity, the DVC amylin and GLP-1 receptors continue to be a viable drug target for sustained weight loss.

## Discussion

Since 2012, four novel compounds have been approved by the FDA for the treatment of obesity^1,46–52^. Among them, liraglutide has proven to be one of the most commonly prescribed pharmacotherapy for treating obesity, due in large part to the pronounced weight loss and relatively modest profile of adverse events^21,22,53^. While all of these approved therapies, including liraglutide, are successful at producing initial weight loss, each is limited in the magnitude, and for some, the duration of efficacy^21,40,54,55^. Indeed, a well-documented phenomenon undoubtedly contributing to the resistance of these drugs in producing a greater duration and magnitude of weight loss is the host of counter-regulatory metabolic and neuroendocrine changes that occur with weight loss^54^. Regardless of the precise mechanisms contributing to this phenomenon, a growing appreciation is that the central nervous system (CNS) is sensing multiple changes in redundant hormonal systems that drive hyperphagia to restore a previous body weight set point^54^. Thus, a theoretical advantage of utilizing combinatorial pharmacotherapy is to provide a pharmaceutical means to counteract even more of these counter-regulatory neuroendocrine changes being sensed by the brain.

Previous reports showed that administration of chimeric peptides of amylin and GLP-1 analogs is effective to induce weight loss in rodents^44^; however, evidence investigating long-lasting and mechanistic effects of combined amylin and GLP-1 pharmacotherapy is still lacking. Results of the current studies support the hypothesis that the combination of amylin with GLP-1-based pharmacotherapy, in particular a stepwise-administration of sCT and liraglutide, results in a greater suppression of feeding, body weight and adiposity compared to administration of amylin or GLP-1 analogs alone.

The pattern of intake suppression and body weight loss in the chronic studies reported here differed over time for the drugs administered individually, combined or in a stepwise fashion. The magnitude of body weight loss for liraglutide-treated rats was stronger than sCT- and vehicle-treated animals. Moreover, while sCT was effective in suppressing body weight for 20-days, it lost its potency during the last 10 days. Monotherapy with liraglutide immediately resulted in a pronounced suppression of body weight, however, the magnitude of weight loss was then stable over the 30-days treatment period. Combination therapy with sCT and liraglutide mimicked the body weight-suppression achieved with liraglutide alone, resulting in a stronger and longer-lasting body weight change during the 3^rd^ and 4^th^ weeks of treatment. Interestingly, stepwise-administration of amylin and GLP-1R analogs overall resulted in the greatest and longest-lasting suppression of food intake and body weight in DIO-rats across the treatment-period. This strongly suggests that stepwise administration should be effective at breaking the weight-loss plateau commonly observed with monotherapies.

Given previous findings that native amylin and GLP-1 control for food intake, in part, through action in the DVC^7,10,26^, raises the intriguing possibility that amylin and GLP-1 receptor analogs might activate a complementary set of partially overlapping neural circuits to affect ingestive behavior. Such a possibility would provide some mechanistic insight to help explain the enhanced and sustained suppression of body weight with a stepwise route of administration. Indeed, our c-Fos analysis clearly demonstrate that treatment with sCT + liraglutide strongly increased the number of DVC-activated neurons, standing out the DVC as an essential hub for mediating the beneficial effect of amylin and GLP-1R analogs combinational therapy. These findings should not be interpreted as an assertion that the DVC is the only CNS site mediating the intake suppressive effects of GLP-1- and amylin-based pharmacotherapy. Indeed, GLP-1R agonists suppress feeding behavior and decreased body weight *via* food-intake-regulatory brain regions including, but not limited to, the hypothalamus and the brainstem^13,56^. Several pieces of evidence demonstrate that GLP-1R agonists target the hindbrain and multiple hypothalamic nuclei (*e*.*g;* paraventricular nucleus of the hypothalamus, dorsomedial hypothalamus, lateral hypothalamus and arcuate nucleus)^57–59^. However, selective knockdown of GLP-1R in the DVC only partially perturbs normal energy balance, whereas GLP-1R knockdown in the hypothalamus does not result in any energy balance impairment^60^. Thus, suggesting that while individually, the DVC and the hypothalamus, appear to be sufficient to mediate the effects of GLP-1R agonists on energy balance, activation of multiple GLP-1R-responsive sites in the brain is necessary for endogenous GLP-1R regulation of feeding.

Another important consideration from the current set of data is that qPCR analyses revealed that sCT and liraglutide, either administered individually, combined or stepwise, did not alter the mRNA expression of amylin and GLP-1 receptors in the DVC. These important negative data support the hypothesis that even in a weight-reduced state from peak obesity, the DVC GLP-1R and amylin receptors theoretically remain as continued viable drug targets for sustaining weight loss. Future studies are certainly warranted to examine amylin and GLP-1 receptor mRNA and protein concentrations in other CNS nuclei. An important limitation that the field as a whole will need to overcome to achieve such analyses will be the development of validated antibodies to not only the GLP-1R but also the CTR and RAMP subunits of the amylin receptor complex.

Of concern, is the novel finding that chronic exposure to HFSD during adulthood, consistently downregulates PPG mRNA expression (but not GLP-1R). These findings suggest that the obese state is potentially able to influence endogenous central GLP-1 signaling. It is worth noting that these findings contrast with conclusions drawn from previous reports^61–63^. Importantly, major differences in the methods and analyses of these previous reports and the current data greatly influence the interpretation and conclusions drawn. In neither Barrera *et al*.^61^ nor Vrang *et al*.^63^, was PPG expression actually quantified. Instead, for both of these aforementioned studies, *in situ* hybridization was employed and few details on anatomical specificity were reported. Further, in the report by Knauf *et al*.^62^, there were no actual differences in the body weights of the mice investigated, completely negating the conclusions of the report. Therefore, based on the current findings showing a downregulation in NTS PPG expression by HFSD diet induced obesity, it is tempting to speculate that this effect might exacerbate or perpetuate a chronic hyperphagic effect that sustains obesity. Exploration of such a hypothesis certainly warrants further investigation.

In summary, current findings show that stepwise administration of the amylin analog sCT with liraglutide results in a sustained and long-lasting body weight loss in obese rats. Furthermore, when the obesogenic status was reduced, the expression of amylin and GLP-1 receptors in the DVC remained stable, suggesting that even in a weight-reduced state form obesity, the DVC GLP-1R and amylin receptors theoretically remain as viable drug targets for sustaining weight loss with continued pharmacotherapy. Data presented here also offer the intriguing possible mechanistic explanation of this long-lasting effect of combined amylin and GLP-1 therapy, by demonstrating an enhanced neuronal activation in DVC by the combination of sCT with liraglutide compared to either mono-therapy. Together with previous reports^12,15,18–20,64–66^, the collective set of data suggest that complementary and partially overlapping neuronal hindbrain circuits act in concert to promote body weight loss. Future studies are certainly warranted to extensively characterize the phenotype and neural anatomical projection fields of the GLP-1-/amylin-activated DVC neurons, as well as the role of astrocytes and microglia^67,68^ in mediating the enhanced intake and body weight suppressive effects of amylin + GLP-1 combinatorial therapy. These studies should also be extended to other nuclei throughout the neuraxis, including those in hypothalamic and mesolimbic reward areas.

## Methods

### Animals

Adult male Sprague-Dawley rats (Charles River Laboratories, USA) were individually housed in double-wide cages with a temperature and humidity-controlled environment on a 12-hour/12-hour light-dark cycle. Rats had *ad libitum* access to water, standard Purina Rodent Chow 5001 (Ralston Purina Company, St. Louis, MO); or animals had access to a free-choice diet of chow and 60% high fat/sucrose diet (HFSD) (Research Diets, Inc., New Brunswick, NJ). To induce obesity (770 g mean body weight at the start of the experiment), rats on Chow + HFSD also received two Nabisco® Golden Oreo cookies per day in addition to *ad libitum* food access. For all the experiments, rats were handled daily and habituated to intraperitoneal (*IP*) injections for one week prior to testing. All procedures were approved by the University of Pennsylvania Institutional Animal Care and Use Committee and conform to the NIH Guide for the Care and Use of Laboratory Animals.

### Drugs

Salmon calcitonin (sCT, H-2260; Bachem), pramlintide (SC-476040; Santa Cruz) and liraglutide (gift of NovoNordisk, Denmark) were dissolved in 0.1 M PBS (pH 7.4) for systemic (IP) injections. Amylin (H-9475.100; Bachem), GLP-1 (7–36; H-67951000, Bachem) and Angiotensin II (Ang II: Bachem) were dissolved in artificial cerebrospinal fluid (aCFS; Harvard Apparatus) for central injection. 5′-bromo-2′-deoxyuridine (BrdU; Sigma) was dissolved in double-distilled water and heated to 40–50 °C.

### Acute systemic administration of combined drug treatments with amylin, GLP-1, sCT and liraglutide

We first aimed to investigate the behavioral mechanism by which combined activation of amylin receptors and GLP-1R suppress food intake in lean rodents using various pharmacological combinations of native amylin with native GLP-1(7–36), as well as combined administration of the amylin receptor/calcitonin receptor agonist, sCT, together with the GLP-1R agonist, liraglutide. These analyses were carried out in two separate groups of rats.

Rats were housed in our automated feedometer system^69,70^, with *ad libitum* access to a food cup resting on a load cell containing powdered chow and water in a hanging wire cage. Food intake was recorded continuously every 10 seconds by computer software (LabView) and meal pattern parameters were calculated. A meal bout is defined as ingestion amounting to at least 0.25 g with a minimum 10-min ingestion-free period between meals. This allowed us to investigate the effects of the combination of native amylin and GLP-1 therapy on the intake suppressive effects in the first meal. Rats (*n* = 14/group) were assigned to receive once-daily *IP* injections at the beginning of the onset of the dark cycle of vehicle (1 ml/kg), amylin (5 μg/kg), GLP-1 (100 μg/kg) or a combination of amylin and GLP-1^10,15,33,65,71–74^.

In separate rats (*n* = 14/group), we examined the combined intake and body weight suppressive effects of long-acting pharmacological analogs of GLP-1R (liraglutide) and amylin (salmon calcitonin; sCT) over the course of 24 h. Rats received an *IP* injection immediately prior to dark cycle onset of either vehicle (1 ml/kg), sCT (3 μg/kg), liraglutide (50 μg/kg) or a combination of both sCT and liraglutide^10,15,33,65,71–74^.

For all experimental groups, treatments were administered in a counter-balanced, within-subject design and injection days were separated by 72 hours.

### Immunohistochemistry

To evaluate neuronal activation in DVC, cFos immunoreactivity was quantified in a separate cohort of rats (315–345 g; *n* = 5 per group) following a 1 h food deprivation during the light phase and a single *IP* injection of either liraglutide (50 μg/kg), sCT (3 μg/kg), liraglutide and sCT (50 μg/kg + 3 μg/kg) or with vehicle (0.1 M PBS). Ninety minutes after drug administration, animals were transcardially perfused using 0.1 M PBS followed by 4% paraformaldehyde (PFA). Brains were collected and maintained overnight in 4% PFA. Subsequently, tissues were transferred to 20% sucrose in PBS for 24 h. Brains were cut into 30-μm coronal sections on a cryostat (Leica microsystem) and stored in cryoprotectant until processing for immunohistochemistry. Free-floating hindbrain sections were collected at the level of the area postrema (from bregma, AP −13.0 to −14.8 mm). Briefly, sections were washed in 50% EtOH and subsequently incubated for 20 min in 1% sodium borohydride. After blocking of endogenous peroxidase, sections were incubated in immunoblocking buffer (5% normal donkey serum and 0.2% Triton-X in PBS) for 1 h, followed by overnight incubation in primary antibody rabbit anti-cFos (1:1000, Cell Signaling, Danvers, USA). Tissues were washed in PBS and incubated for 2 h with fluorescent secondary antibody Alexa Fluor 594 donkey anti-rabbit (1:500, Jackson ImmunoResearch, USA). Then, sections were rinsed in PBS and mounted onto Superfrost Plus glass slides (Thermo Scientific, Waltham, MA USA) and coverslipped with DAPI mounting medium. cFos-positive cells in the NTS, AP and DMV were quantified from DVC containing sections and averaged within nuclei for each animal from bregma, AP −13.0 to −14.8 mm. Counts for each nucleus were then averaged across animals per treatments to determine the neural activation in the DVC elicited by liraglutide, sCT or the combination of liraglutide and sCT compared to vehicle. Counting was accomplished at 20X magnification and numerical aperture of 0.75, using a light/fluorescent microscope (Nikon Eclipse Ni, Nikon, USA) and Imaris software version 8.1.2 (Bitplane, Switzerland).

### Chronic daily administration of an amylin receptor or GLP-1R analog as a mono- or combinatorial-therapy on body weight gain and food intake

Experiments were designed to determine whether chronic combinatorial administration of an amylin receptor agonist with a GLP-1R agonist is superior in producing a greater magnitude of sustained weight loss and food intake suppression compared to monotherapy in a diet induced obese rat model. These studies were also designed to determine whether the typical plateau of weight loss that occurs with any pharmacotherapy intervention^22,23,40,41,46,52,54,75^, even combinatorial therapies^33,50,51^ could be overcome through a unique stepwise approach of introducing the combinatorial therapy in attempt to overcome the tachyphylaxis of sustained pharmacotherapy administration from the beginning of treatment. To this end, obese rats (*n* = 10–12 per group; matched for mean body weight at the start of treatment at 770 g ± 10 g) received daily *IP* injections of vehicle (1 mL/kg), sCT (3 μg/kg), liraglutide (50 μg/kg), a combination of sCT and liraglutide (3 μg/kg + 50 μg/kg) or a stepwise administration of the amylin and GLP-1R analogs, where rats received sCT treatment for the first week, liraglutide treatment for the second week, followed by a combination treatment of sCT and liraglutide for the remaining testing period. Injections were given 15 minutes prior to the onset of the dark cycle and body weight and food intake were recorded daily or every 48 h respectively, through the ~9 weeks of experimentation. These procedures were run using two cohorts of rats, with identical timing and methods between cohorts and all groups represented in each cohort. Food intake was recorded every 48 h (accounting for crumb spillage) beginning at dark cycle onset.

### Post-mortem tissue and plasma collection

At the completion of behavioral testing, all rats were deeply anesthetized with a cocktail of ketamine (90 mg/kg), xylazine (2.7 mg/kg) and acepromazine (0.64 mg/kg) and decapitated. Brains were immediately removed and flash-frozen in cold isopentane to preserve RNA. Trunk blood was collected in chilled heparinized vacutainer tubes for measuring circulating leptin and insulin levels. Plasma was stored at −80 °C until ELISA assay. Plasma leptin and insulin were measured using commercial kits (Millipore, St. Louis MO) according to manufacturer’s instructions. A calibration curve and an internal control were included in each plate. Finally, inguinal, retroperitoneal, epididymal, and perirenal white fat depots were removed and weighed.

### RNA extraction and quantitative real-time PCR (qPCR)

After decapitation, brains were removed, snap-frozen in −70 °C isopentane and stored at −80 °C until processing. NTS-enriched micropunches were taken at the level of the AP (from bregma, AP −13.0 to −14.8 mm) and RNA was extracted according to manufacturer’s instructions (TRI Reagent; Sigma Aldrich) and then purified following the cleanup protocol of RNeasy Mini kit (Qiagen), similar to our previous reports^65,76,77^. The concentration and the integrity of RNA were measured using a nanodrop system (NanoDrop 1000 Spectrophotometer, Thermo Scientific, Waltham, MA, USA).

Total RNA was reverse transcribed with Advantage RT for PCR Kit (Clontech, USA). Real-time PCR was performed using 7500 Fast system (Applied Biosystem/Life Technologies Carlsbad, CA, USA) with Taqman Gene Expression Master Mix kit (Applied Biosystem, USA). Quantitative polymerase chain reaction was performed using pre-designed Taqman probes (Thermoscientific, USA) for rat GAPDH (Rn01775763), GLP-1R (Rn00562406_m1), CTR (Rn01526770_m1), RAMP1 (Rn01427056_m1), RAMP2 (Rn00824652_g1), RAMP3 (Rn00571815_m1), BDNF (Rn02531967_s1) and GDNF (Rn01402432_m1) transcripts. Intron-spanning primers for PPG were designed with IDT (Integrated DNA Technologies). The primer sequences were the following: sense: 5′-ACCGCCCTGAGATTACTTTTCTG-3′, antisense: 5′-AGTTCTCTTTCCAGGTTCACCAC-3′ and probe: 5′-6-FAM™-CGCAGTCACGCAACCTGGATTACAA-ZEN-3′ (Iowa Black® FQ). qPCR conditions were: initial heat activation at 95 °C for 10 min, followed by 40 cycles of alternating between 95 °C for 15 s, and 60 °C for 60 s. The fold change in expression of each gene was calculated using the comparative DDCt method, with GAPDH as housekeeping reference endogenous control. Each sample was run in duplicate.

### Statistical analysis

All data were entered in Excel version 16.10 and subsequently subjected to statistical analysis using GraphPad Prism Software version 7.0 (San Diego, CA, USA). Statistical significance was set at P < 0.05. Statistical evaluation of the data was carried out using Student t-test, 1- or 2- way ANOVA with Tukey post-hoc analysis (One-way) or Sidak post-hoc analysis (Two-way) between control and treatment groups in cases in which statistical significance was established. All data are expressed as mean ± S.E.M.
